# Challenges in the Diagnosis and Management of Giant Porokeratosis: A Case Report

**DOI:** 10.7759/cureus.55155

**Published:** 2024-02-28

**Authors:** Mariana Georgiana Portelli, Beatrice Bălăceanu-Gurău, Olguta Anca Orzan, Sabina Andrada Zurac, Irina Tudose

**Affiliations:** 1 Department of Dermatology, Elias Emergency University Hospital, Bucharest, ROU; 2 Department of Dermatology, Carol Davila University of Medicine and Pharmacy, Bucharest, ROU; 3 Department of Pathology, Carol Davila University of Medicine and Pharmacy, Bucharest, ROU; 4 Department of Pathology, Elias Emergency University Hospital, Bucharest, ROU

**Keywords:** genodermatosis, premalignant condition, keratinization, cornoid lamella, giant porokeratosis, porokeratosis of mibelli, porokeratosis

## Abstract

Porokeratosis encompasses a diverse group of dermatoses, both acquired and genetic, marked by a keratinization disorder. Porokeratosis of Mibelli (PKM) presents as solitary plaques or multiple papules/macules with central atrophy and raised hyperkeratotic borders. Here, we present a case of giant porokeratosis (GPK), a rare form often considered a morphological variant of PKM, with unique clinical and histopathological aspects. Our case involves a 29-year-old patient with a 15 × 10 cm irregular plaque on the dorsal aspect of the right hand. The patient was previously prescribed various topical treatments (retinoids, calcineurin inhibitors, and combinations of corticosteroids with vitamin D3 analogs) and systemic retinoids without improvement before presenting to our department. Due to the high risk of neoplastic transformation and the unavailability of imiquimod, the patient was recommended topical 5-fluorouracil treatment. The trajectory of the lesion under treatment revealed a favorable evolution, and the patient was subjected to regular monitoring every three months to assess the ongoing progress. Recognizing GPK as a high-risk variant is crucial for dermatologists, and it requires a personalized approach. Regular monitoring is advised to detect potential malignant transformations promptly. Future research holds promise for diagnostic advancements, refined treatment modalities, and a deeper understanding of the molecular mechanisms underlying malignancy in porokeratosis.

## Introduction

Porokeratosis is a diverse group of dermatoses, either acquired or genetic, characterized by a keratinization disorder with a clinical presentation of an atrophic center surrounded by a hyperkeratotic border and a histological presence of the cornoid lamella [1,2].

Recent studies suggest a possible reclassification of porokeratosis as genodermatosis, given its association with heterozygous germline mutations, often in genes encoding enzymes of the mevalonate pathway. Despite being traditionally categorized as a keratinization disorder, the underlying genetic factors indicate a broader genodermatosis perspective [3].

The term porokeratosis was introduced by Vittorio Mibelli in 1893, being derived from the Greek words “poro” meaning pore (of the sweat gland) and “keratosis” meaning horny thickening [2].

Since the initial reports by Neumann in 1875 and later by Respighi and Mibelli in 1889, numerous subtypes of porokeratosis have been introduced based on distinct morphology, distribution, and histopathology. These include disseminated superficial actinic porokeratosis (DSAP), the most prevalent variant, porokeratosis of Mibelli (PKM), the second most common variant, disseminated superficial porokeratosis, and palmoplantar porokeratosis (encompassing porokeratosis palmaris, plantaris et disseminata, punctate porokeratosis, linear palmoplantar porokeratosis, porokeratotic palmoplantar keratoderma discrete, spiny keratoderma, porokeratosis palmaris discrete variants). Additional clinical variants have also been reported, and it remains unclear whether some of these entities represent subvariants of the main six forms or distinct entities [4].

PKM is a rare and chronic skin condition presenting as a single, centrifugally spreading solitary plaque or multiple papules/macules with central atrophy and raised keratotic borders, typically unilateral, less frequently bilateral, or symmetric, varying in size of up to 20 cm. Various subtypes, including linear/neviform, giant, and hyperkeratotic/verrucous forms, have been observed. While PKM predominantly appears on the limbs, particularly hands and feet, it can also manifest on the face, lips, palms, soles, genitalia, scalp, and oral mucosa. Although usually asymptomatic, PKM may be pruritic. PKM exhibits a male predominance and can be either sporadic or familial, transmitted as an autosomal dominant trait with variable penetrance. In familial cases, lesions typically emerge during childhood, increasing in size or number over the years [2,4].

Multifactorial etiopathogenesis postulates that in porokeratosis, a mutant clone of epidermal cells expands peripherally, forming a cornoid lamella at the boundary between the clonal population and normal keratinocytes. This tendency for abnormal clones is likely inherited, with additional triggering factors such as ultraviolet light, photochemotherapy, irradiation, infective agents, mechanical trauma, and immunocompromised states leading to clinical manifestation. An alternative hypothesis suggests that an inflammatory mononuclear infiltrate beneath the cornoid lamella may stimulate overlying keratinocytes [5-7].

Some studies suggest that this abnormal keratinocyte proliferation may result from the loss of immunosurveillance, associations being made with immunodeficiencies, organ transplantation, leukemias/lymphomas, human immunodeficiency virus infection, and inflammatory diseases treated with immunosuppressive drugs, including biological therapies. This may occur by impeding the identification and/or elimination of abnormal keratinocyte clones by immune cells, potentially contributing to the generation and expansion of a population of mutant keratinocytes. The development of porokeratosis on burn scars or hemodialysis access regions suggests an isomorphic phenomenon, but the reproduction of this finding was unsuccessful [2].

Dermoscopy, an essential diagnostic tool, offers valuable insights into porokeratosis. PKM exhibits distinctive features, including a white peripheral keratotic rim and linearly arranged brown clods, often with fewer blood spots and erosions than DSAP. The furrow ink test, done by staining the skin surface with a whiteboard marker, aids in diagnosis by emphasizing rims and open pores [4].

Regarding pathology, a biopsy must encompass the peripheral rim of the lesion. The presence of the cornoid lamella serves as a distinctive histopathological marker across all porokeratosis subtypes. It exhibits a stack of tightly fitted parakeratotic cells, clearly demarcated from the surrounding corneocytes, extending through the stratum corneum. Beneath the column of parakeratotic cells, the granular cell layer is typically absent, leading to a thinned epithelium. Another characteristic feature in PKM includes epidermal invagination with papillomatosis in the central part of the lesion. This is often accompanied by hyperkeratosis, irregular acanthosis, upper and mid-dermal lymphohistiocytic inflammatory infiltrate, and the presence of perivascular plasmocytes [4,6].

## Case presentation

**Figure 1:**
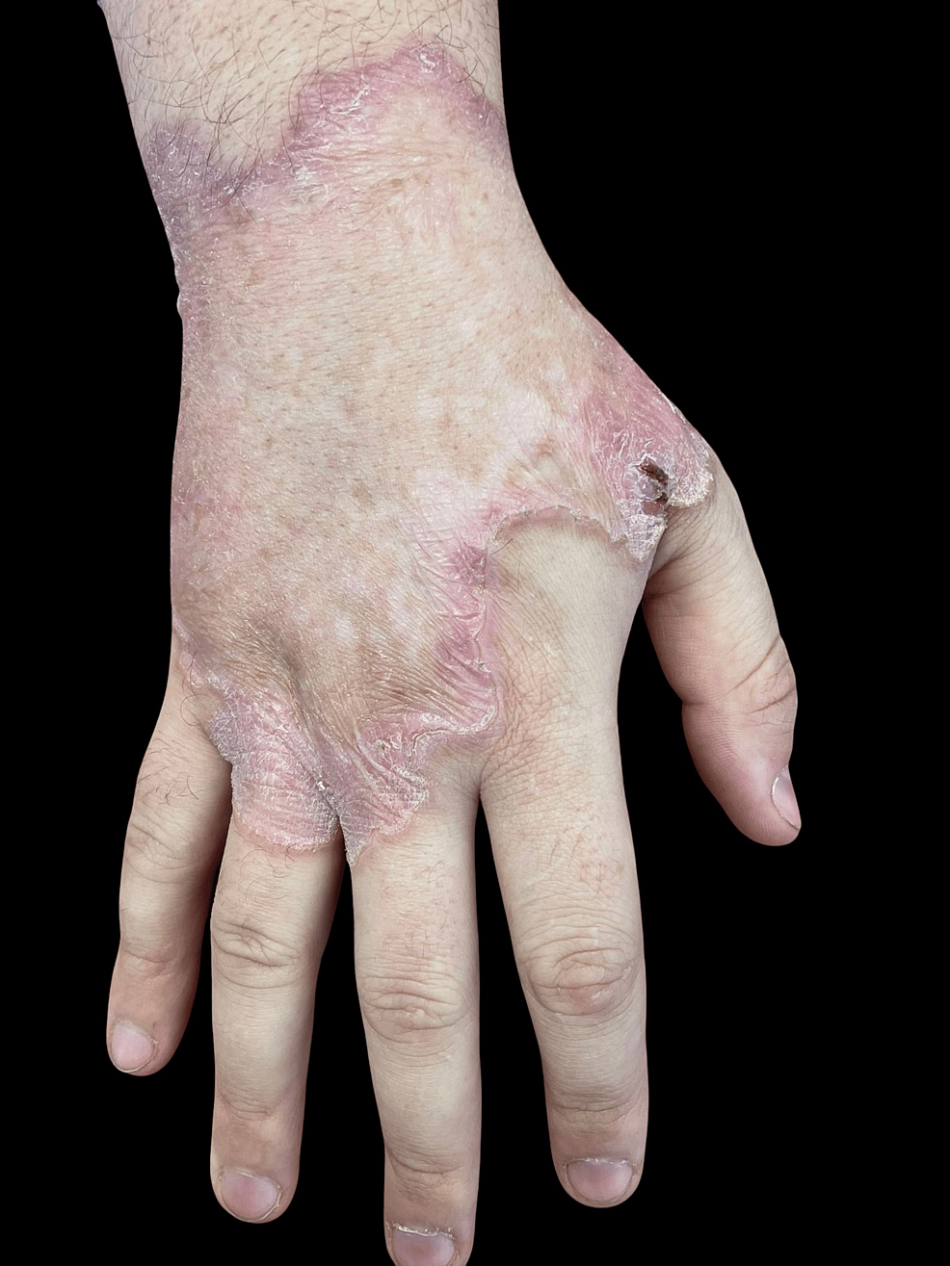
Giant plaque of porokeratosis surrounded by a raised hyperkeratotic border and an atrophic center.

We present a case involving a 29-year-old patient with an irregular plaque measuring approximately 15 × 10 cm in diameter. The lesion displayed an atrophic center surrounded by a raised hyperkeratotic border, reaching 6-7 mm in height, and was situated on the dorsal aspect of the right hand (Figure 1). The patient reported sensations of stinging, pain, and itching at the lesion site, exacerbated by exposure to sunlight. He had no comorbidities and lacked a familial history indicative of analogous afflictions.

Starting 19 years prior as a small keratotic papular lesion, the condition was initially misdiagnosed as verruca vulgaris, considering the patient’s age and clinical presentation. Initial treatment involved the application of a common celandine-based cream. Subsequently, the lesion exhibited gradual progression without periods of remission.

He consequently applied topical combinations of antibiotics and corticosteroids which failed to yield any clinical improvement. The first biopsy conducted at the age of 15 showed hyperplastic epithelium with parakeratotic columns in epidermal invaginations, the absence of the underlying granular layer, and keratinocytes irregularly arranged beneath the parakeratotic transformation.

Following the diagnosis of PKM through clinicopathological correlation, systemic retinoid treatment with acitretin 30 mg daily was prescribed. Unfortunately, the course was discontinued after seven weeks because of significant xerosis and arthralgias, with only marginal improvement. The patient also underwent other topical treatments, including retinoids (tretinoin 0.02%) calcineurin inhibitors (tacrolimus 0.1%), and combinations of corticosteroids with vitamin D3 analogs (betamethasone dipropionate 0.064% + calcipotriene 0.005%) for short periods, none of which yielded significant or sustained improvement.

**Figure 2:**
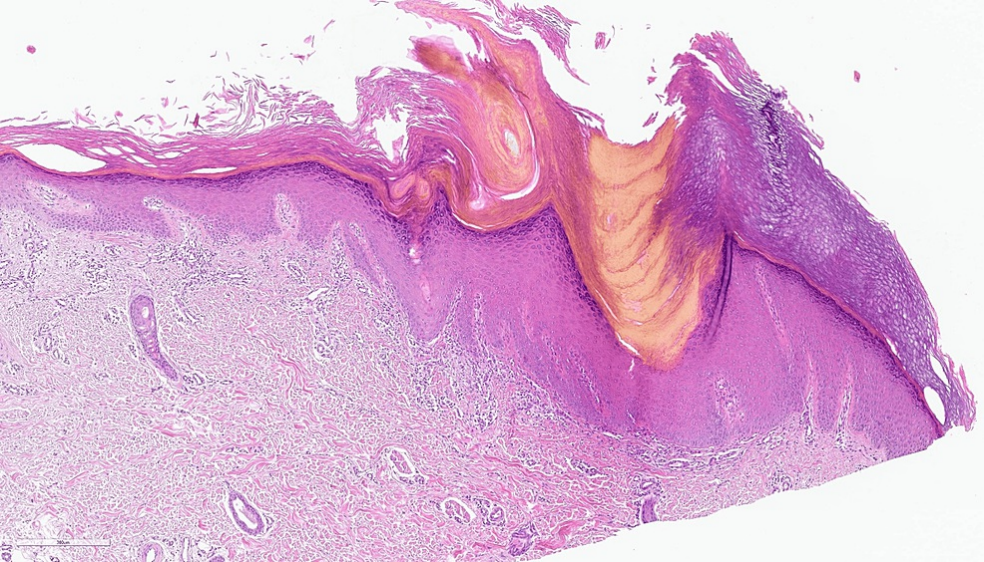
Hyperkeratotic lesion with mild parakeratosis, dermal lymphocytic infiltrate, and dilated capillaries in the papillary dermis (hematoxylin and eosin staining, 40X)

**Figure 3:**
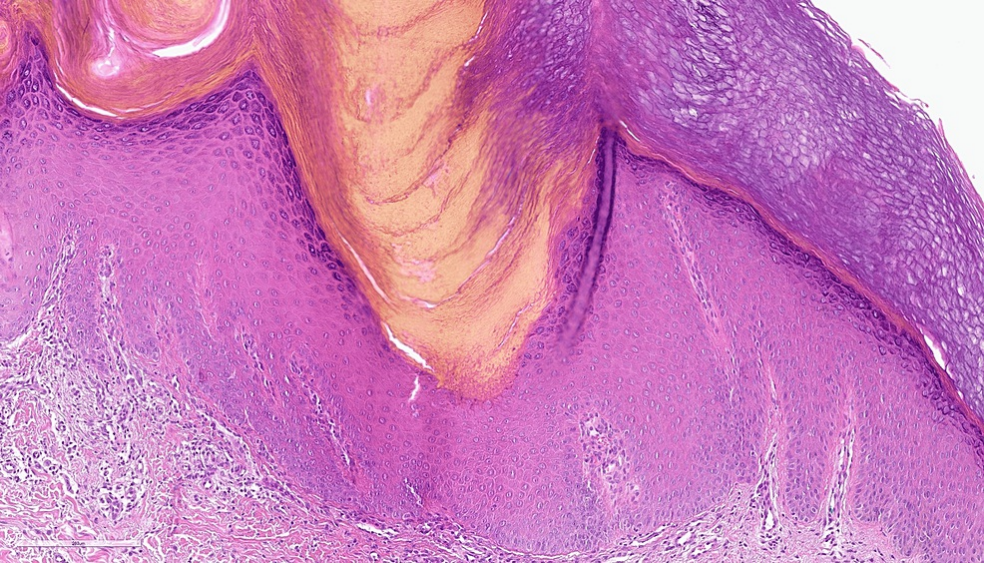
Parakeratotic column overlying a small vertical zone of dyskeratotic and vacuolated cellule within the epidermis (cornoid lamella), and loss of the granular layer (hematoxylin and eosin staining, 200X)

A subsequent biopsy at the age of 24 revealed moderate hyperortokeratosis on the surface, parakeratosis with a vertical, angled arrangement, overlying an area of invagination of the epidermis with corresponding agranulosis (cornoid lamella appearance) (Figures 2, 3).

**Figure 4:**
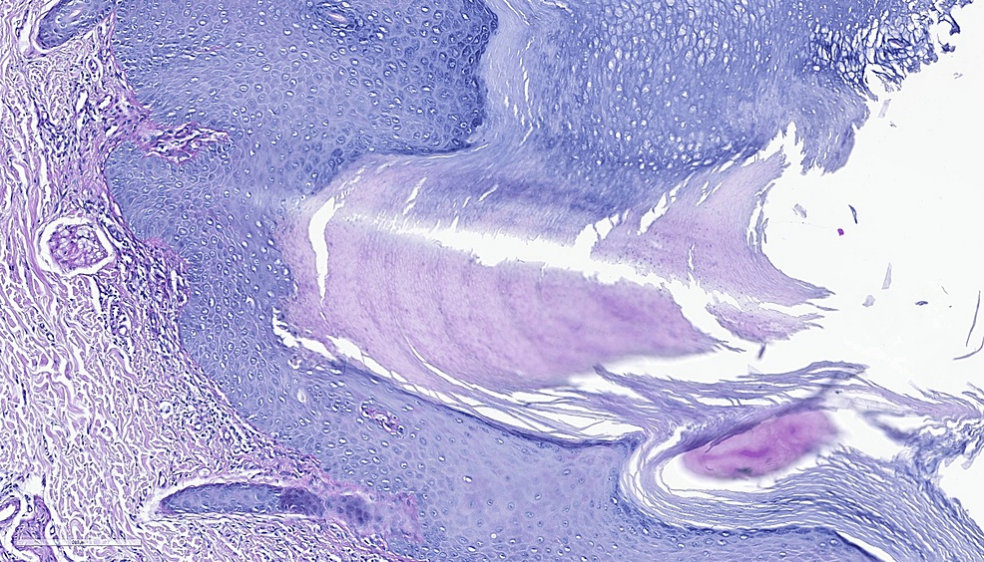
Intact basal membrane on periodic acid-Schiff staining (100X).

**Figure 5:**
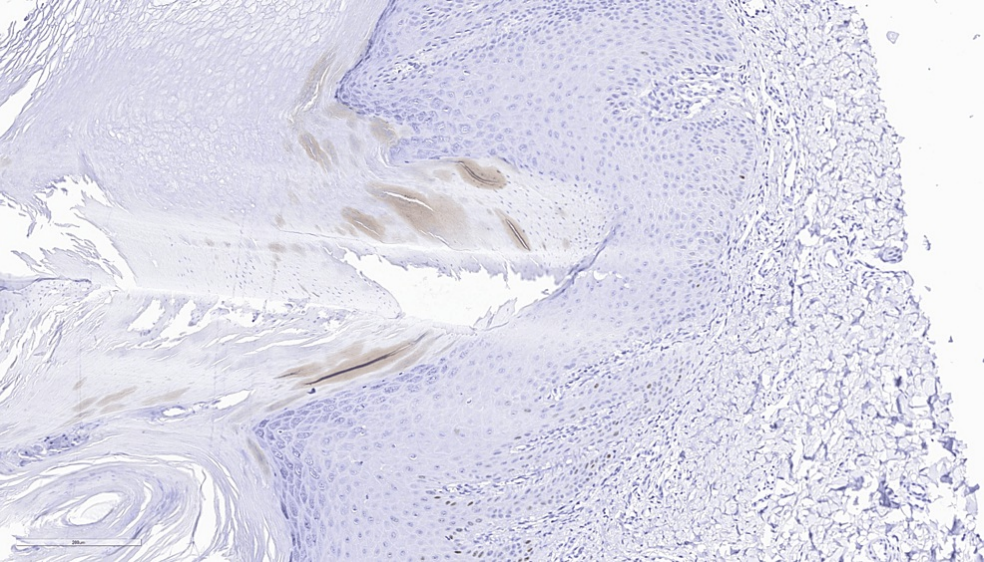
The immunohistochemistry image shows positive p53 in the nuclei of basal keratinocytes (200X).

Additional findings comprised moderate, relatively uniform acanthosis, focal vacuolar basal degeneration, rare colloidal bodies in the basal layer and papillary dermis, moderate perivascular lymphocytic inflammatory infiltrate, and a slightly prominent superficial vascular plexus with capillaries featuring swollen endothelium. The histopathological findings were consistent with porokeratosis, ruling out neoplastic degeneration (Figures 4, 5).

Upon consultation in our service, the patient was advised to use 5% 5-fluorouracil (5-FU) cream due to the high risk of neoplastic transformation, particularly in giant porokeratosis with an evolution period of approximately 20 years. Surgical excision was deemed unfeasible owing to the lesion’s substantial size, anatomical location, and patient reluctance. The absence of imiquimod on the Romanian market at the time further supported the selection of topical 5-FU 5% cream, a decision substantiated by favorable outcomes reported in the literature for similar cases. At the initial assessment, the patient’s progress was deemed slowly favorable, necessitating a follow-up every three months.

## Discussion

Giant porokeratosis (GPK), a very rare form, is suggested by some reports as a morphological variant of PKM, while others consider it a distinct clinical form. While there is no strict size definition, most consider GPK to be at least 10-20 cm in diameter. It clinically resembles PKM, featuring an atrophic center surrounded by a hyperkeratotic border raised up to 1 cm. Given that the most extensively documented porokeratosis lesion measured 25 x 50 cm, it is imperative to underscore the importance of considering distinct anatomical locations when ascribing the term “giant” to porokeratosis [1,5,8].

Malignant transformation to Bowen’s disease, squamous cell carcinoma, and basal cell carcinoma is a documented occurrence in all forms of porokeratosis, with reported incidences ranging from 7.5% to 11% [2,8]. Linear porokeratosis carries the highest risk, followed by GPK. Various risk factors, such as large lesion size, extremity location, and extended duration of existence, contribute to the increased oncogenic potential. The average time until the onset of cutaneous neoplasia in porokeratosis was assessed to be approximately 33.5 years [2]. In this instance, the patient exhibited potential risk factors, including prolonged duration, considerable size, extremity location, and the absence of suitable therapeutic interventions with proven efficacy. Early intervention is strongly recommended for the optimal management of GPK, preventing dysplasia and the development of neoplasia [1,6,8].

For numerous patients, safeguarding against sunlight, using moisturizers, and conducting regular examinations for malignancies may suffice. In instances where lesions are extensive and medical intervention is sought, several treatment options have been suggested. Owing to the absence of comprehensive placebo-controlled trials, the utilization of therapeutic modalities has predominantly occurred in singular cases or small case series. No treatment has shown consistent and long-lasting efficacy [2,6]. The choice of an optimal treatment approach depends on factors such as lesion size, location, and functional and aesthetic considerations [5].

The patient presented in this case report employed a range of treatments, including topical steroids, topical and systemic retinoids, calcineurin inhibitors, and vitamin D3 analogs. While topical steroids can provide temporary relief, caution is advised due to the potential for immunosuppression-induced porokeratosis proliferation and malignant transformation. Retinoids modulate epidermal turnover and accelerate keratinocyte proliferation. Although systemic retinoids, such as etretinate, isotretinoin, or acitretin, have demonstrated success and superiority over topical retinoids in addressing cytological atypia and halting carcinogenesis, prolonged use is limited by side effects. Reports indicate varying outcomes with topical treatments, with some combinations yielding partial improvements and others proving ineffective. While tacrolimus 0.1% ointment has been documented to induce enduring clearance in a patient afflicted with linear porokeratosis, its efficacy in the context of PKM has not been prominently acknowledged. Vitamin D derivatives, including calcipotriol cream 0.005%, have been tested in a limited patient population with DSAP, with positive outcomes after extended applications ranging from 6 weeks to 19 months; however, it is noteworthy that their application in PKM has not been documented [2,4].

Various therapeutic modalities have been explored for the treatment of porokeratosis, including surgical excision (shaving), which proves effective for patients with a limited number of lesions when free excision margins are obtained, though it may lead to the development of hypertrophic scars, contractions, post-surgical neuralgia, and recurrence. Cryotherapy/cryosurgery has demonstrated efficacy in some cases, particularly with prolonged freezing cycles, although extended treatment may be required for clearance, and it poses risks of hyperpigmentation, scarring, or atrophy. Laser ablation or vaporization, utilizing erbium, CO_2_, or Q-switched ruby lasers, has produced mixed results, with potential complications such as scars and secondary hypopigmentation. Photodynamic therapy (PDT) has been employed in PKM, either as a standalone method or in conjunction with topical treatments. MAL-PDT, involving one to four sessions, demonstrated moderate-to-excellent results. Combined therapy with 5% 5-FU daily and ALA-PDT achieved complete clearance by week three, with no recurrence at the six-month follow-up [2,4].

Topical 5% 5-FU cream has been utilized in combination with other treatments for porokeratosis. In a PKM case, a daily application under occlusion for eight weeks resulted in 30% resolution, with a notable inflammatory reaction. Subsequent treatment with topical 5% imiquimod (three days per week for six weeks) achieved complete healing. A combined topical therapy (morning 5% 5-FU and evening 5% imiquimod) initially showed inefficiency by week four, but, with modification, led to complete remission at week 12. Conversely, cases treated with 5% 5-FU every other day for six to eight weeks, along with an unknown dosing regimen, showed no response or incomplete responses. In another PKM case, an eight-week therapy with 5% 5-FU cream (unknown regimen) improved the lesions. Other combination approaches, such as 5-FU with a 70% glycolic acid peel delivered in pulse doses, and 5-FU combined with PDT, have also been explored [2,4].

Imiquimod 5% cream has been successful in treating cases of PKM, with an application frequency of three to seven days per week for 4-24 weeks. Notably, inflammation often developed, sometimes to an unacceptable degree, with lesions regressing but leaving residual scarring or hypopigmentation [2,4].

Alternative local treatments mentioned in the literature encompass keratolytic agents (urea, salicylic acid), cantharidin, diclofenac gel, and ingenol mebutate gel [2,4].

Given the documented success of 5% 5-FU treatment in reported cases within the literature, coupled with the observed inefficacy of prior treatments and the unavailability of imiquimod, 5-FU was recommended to our patient.

## Conclusions

In summary, the recognition of GPK as an infrequent yet high-risk variant is paramount for dermatologists. The management of porokeratosis necessitates a personalized and meticulous approach, incorporating considerations of lesion dimensions, anatomical location, functional and aesthetic implications, malignancy risk, and patient preferences. Regular monitoring is advisable for patients with prolonged and sizable porokeratosis lesions to promptly identify any potential malignant transformations.

The future trajectory of porokeratosis research holds promising avenues for advancements in diagnosis, monitoring, and treatment. Non-invasive imaging techniques are anticipated to refine diagnostic precision, while large placebo-controlled trials will contribute robust evidence of the efficacy of diverse treatment modalities. Simultaneously, research endeavors are poised to delve into the molecular intricacies of malignant transformation, employing longitudinal studies to discern risk factors for malignancy development. These will undoubtedly shape a more comprehensive and nuanced understanding of porokeratosis, paving the way for improved patient care and outcomes.
